# Anxiety prevalence among women with polycystic ovary syndrome in mainland China: a systematic review and meta-analysis

**DOI:** 10.3389/fpsyg.2026.1767567

**Published:** 2026-03-11

**Authors:** Shanshan Hong, Zhenzhen Hong, Liying Chen, Meiling Liang, Ming Li, Jiawei Qin

**Affiliations:** 1 Department of Obstetrics and Gynecology, Quan Zhou Women’s and Children’s Hospital, Quanzhou, China; 2 Department of Endocrinology, Quanzhou First Hospital Affiliated to Fujian Medical University, Quanzhou, China; 3 School of Basic Medical Sciences, Hunan University of Medicine, Huaihua, China; 4 Department of Rehabilitation Medicine, Quanzhou First Hospital Affiliated to Fujian Medical University, Quanzhou, China

**Keywords:** anxiety, China, meta-analysis, polycystic ovary syndrome, prevalence

## Abstract

**Objective:**

This systematic review and meta-analysis aimed to determine the pooled prevalence of anxiety among women with polycystic ovary syndrome (PCOS) in mainland China and explore the potential contributors related to anxiety prevalence among PCOS.

**Methods:**

We searched PubMed, EMBASE, Web of Science, CNKI, WanFang, and VIP databases from inception to January 2025. Pooled prevalence estimates were calculated using random-effects models, with subgroup analyses stratified by region, age, BMI, assessment tools, and diagnostic criteria. Heterogeneity was assessed by *I*^2^ statistics and meta-regression.

**Results:**

Thirty-five studies (8,655 participants) met inclusion criteria. The pooled anxiety prevalence was 32% (95% CI: 26–38%), with substantial heterogeneity (*I*^2^ = 97.8%, *p* < 0.001). Subgroup analyses revealed higher prevalence in younger women (<26 years: 39% vs. ≥26 years: 25%), West China (36% vs. East/Central China: 28%), and studies using GAD-7 (41% vs. SAS: 31%). Diagnostic criteria influenced estimates (Rotterdam: 31% vs. PRCHIS: 27%). Meta-regression identified no significant moderators. Sensitivity analyses confirmed robustness, and publication bias was nonsignificant.

**Conclusion:**

Anxiety affects nearly one-third of Chinese women with PCOS, with prevalence shaped by age, region, and assessment methodologies. Despite alignment with global trends, regional disparities underscore the need for culturally adapted mental health interventions. Clinicians should prioritize anxiety screening in PCOS management, particularly for younger women and underserved populations.

## Introduction

Polycystic ovary syndrome (PCOS), a prevalent endocrine disorder affecting 6–20% of women globally, is characterized by hyperandrogenism, ovulatory dysfunction, and polycystic ovarian morphology (Rotterdam, 2004; Joham et al., 2022). In China, its prevalence ranges from 5.6 to 10%, with significant regional variations linked to different diagnostic criteria and participant characteristics (Wu et al., 2021; Li et al., 2013). Beyond reproductive complications, PCOS is associated with metabolic disturbances, including insulin resistance, obesity, and cardiovascular risk, contributing to long-term morbidity (Ni et al., 2009; Aziz et al., 2012). Emerging evidence highlights its profound psychosocial burden, particularly anxiety disorders, which impair quality of life and exacerbate physical symptoms (Yin et al., 2021).

Anxiety, marked by excessive worry and physiological hyperarousal, is highly prevalent in chronic conditions, yet its intersection with PCOS remains underexplored in Chinese populations. The bidirectional relationship between PCOS and anxiety remains understudied (Farkas et al., 2014). Hyperandrogenism and chronic inflammation, hallmarks of PCOS, may dysregulate hypothalamic–pituitary–adrenal axis activity, fostering neuroendocrine pathways that predispose to anxiety (Cooney and Dokras, 2017). Emerging evidence suggests that women with PCOS face unique psychosocial stressors, including infertility concerns, body image dissatisfaction, and societal pressures linked to traditional gender roles, which may amplify anxiety risk (Sari and Gungor Satilmis, 2024). Conversely, anxiety exacerbates insulin resistance and unhealthy behaviors, perpetuating metabolic dysfunction (Lim et al., 2019). Anxiety may lead to an increase in the secretion of stress hormone cortisol, affecting the inflammatory process and exacerbating endocrine disorders in PCOS patients (Stener-Victorin et al., 2024). PCOS is increasingly conceptualized through a biopsychosocial framework, recognizing it as a complex multisystem disorder with profound neuropsychological implications beyond its reproductive features (Teede et al., 2023; Dokras, 2025). Clinical manifestations such as hyperandrogenism-induced physical changes and infertility risks contribute to a distorted body image and chronic stress, which serve as critical precursors to anxiety (Cooney and Dokras, 2017). In China, these psychological burdens are often exacerbated by cultural emphasis on reproductive success and esthetic standards, necessitating a focused investigation into anxiety prevalence. Cultural factors unique to China—such as stigma surrounding infertility and societal pressures for marriage—may further amplify psychological distress in PCOS patients (Wu et al., 2024).

Anxiety symptoms were very prevalent in women with PCOS, although the prevalence varied depending on the self-reported questionnaire used: the Hamilton Anxiety Scale 69.4%, the Hospital Anxiety and Depression Scale 41.5%, and the Self-rating Anxiety Scale 32.4% (Infante-Cano et al., 2024). The prevalence of hypertension, renal disease, gastrointestinal disease, eating disorders, mental illness, depression-anxiety, rheumatoid arthritis, respiratory infections, and all malignancies were 20–40% higher in those with PCOS, compared to controls (Vine et al., 2024). The excess economic burden attributable to PCOS globally is enormous, the additional direct healthcare costs associated with anxiety in PCOS were estimated to be $1.939 billion/year in US (Yadav et al., 2023). Despite global meta-analyses reporting elevated anxiety prevalence in PCOS (Wang et al., 2021), which predominantly focus on Western populations, with limited inclusion of Chinese studies (Nasiri-Amiri et al., 2023). Some studies only showed that the incidence of anxiety in patients with PCOS was higher than that in healthy control group, but did not report the prevalence of anxiety in patients with PCOS (Blay et al., 2016), let alone explore the difference in prevalence among different characteristic groups (Wang et al., 2021). Such issues underscore the need for a systematic synthesis of data to clarify the true burden of anxiety in Chinese population.

This systematic review and meta-analysis aims to fill critical gaps by conducting the first meta-analysis of anxiety prevalence among women with PCOS in mainland China. This work addresses urgent calls from the International PCOS Network to prioritize mental health research in understudied regions (Hoeger et al., 2021). We will explore subgroup variations by region, age, BMI, diagnostic criteria, and assessment tools, while addressing methodological heterogeneity and publication bias. This systematic integration of current findings will inform the development of standardized mental health assessments and proactive management strategies, aiming to alleviate the socioeconomic and clinical impacts linked to comorbid anxiety in Chinese women diagnosed with polycystic ovary syndrome.

## Method

This systematic review and meta-analysis adhered to the Preferred Reporting Items for Systematic Reviews and Meta-Analyses (PRISMA) guidelines. As the study exclusively analyzed deidentified data from published literature, institutional review board approval and informed consent were not required.

### Literature search strategy

A comprehensive search of international (PubMed, EMBASE, Web of Science) and Chinese (CNKI, WanFang, VIP) databases was conducted from database inception through January 8, 2025. The search strategy combined Medical Subject Headings (MeSH) and free-text terms encompassing three domains: (1) anxiety disorders, (2) polycystic ovary syndrome, and (3) geographic focus (“China,” “Chinese population”). No language restrictions were applied, though non-human studies were excluded. Conference abstracts meeting inclusion criteria were tracked for subsequent full-text publications, with direct investigator contact initiated for unavailable manuscripts. To minimize selection bias, backward citation tracking of included articles was performed during full-text screening. Detailed search syntax for all databases is provided in Supplementary Table 1. All identified records were managed using EndNote X9 (Clarivate Analytics), with automated duplicate removal followed by manual verification.

### Study inclusion and exclusion criteria

Studies were included if they met the following conditions: (1) Condition: The study involved women with a confirmed diagnosis of polycystic ovary syndrome (PCOS), based on established diagnostic criteria such as the Rotterdam criteria; (2) Context: The study reported the prevalence of anxiety among women with PCOS or provided sufficient data to calculate this prevalence. Eligible designs included cross-sectional, retrospective, or prospective cohort studies, each with at least 30 participants. Only studies that used validated tools for diagnosing anxiety were considered; (3) Population: Participants were women from mainland China, excluding Hong Kong, Macau, and Taiwan; (4) Duplicate Articles: If multiple studies used the same participant sample, only the most comprehensive article was included.

Studies were excluded if they met any of the following criteria: (1) Condition: Studies that examined the prevalence of PCOS in individuals primarily diagnosed with anxiety; (2) Context: Case reports, reviews, meta-analyses, letters, or study protocols were excluded. Additionally, studies where PCOS diagnosis was self-reported were not considered; (3) Population: Studies that assessed anxiety only in relation to specific PCOS symptoms, such as obesity, infertility, or hirsutism, were excluded. Pregnant women with PCOS were also excluded. Disagreements in the inclusion/exclusion process were resolved through consultation with a third reviewer.

### Study selection, data extraction, and quality assessment

Duplicates were removed, and two blinded reviewers independently screened the titles and abstracts, followed by a full-text assessment to identify relevant studies. Data were extracted using a standardized form, which captured key information, including the authors, publication year, language, study location, survey period, participant demographics (age, BMI), anxiety screening tool, diagnostic criteria for PCOS, sample sizes for both anxiety cases and PCOS cases, and the prevalence of anxiety, when available. In cases of missing or unclear data, the corresponding authors were contacted.

The risk of bias for each included study was independently evaluated by two reviewers using the 10-item checklist developed by Hoy et al., which is specifically validated for prevalence studies (Hoy et al., 2012). This tool assesses two primary domains: external validity (Items 1–4: focusing on the representation of the target population, sampling frame, random selection, and non-response bias) and internal validity (Items 5–10: focusing on data collection methods, case definition, instrument validity, consistency in data collection, and appropriate prevalence parameters). Each item was assigned a score of 1 (‘Low risk’) or 0 (‘High risk’). An 11th item was included to provide an overall summary of bias. Based on the cumulative scores, the studies were categorized as having a low risk (scores > 8), moderate risk (scores 6–8), or high risk of bias (scores ≤ 5). Any discrepancies between the two reviewers were resolved through consensus or by consulting a third senior investigator.

### Statistical methods

Analyses were implemented in STATA/SE 15.0 (StataCorp, College Station, TX, United States). We calculated pooled prevalence estimates of anxiety with 95% CIs using random-effects models. Heterogeneity was assessed using the I^2^ statistic (values >75% indicating substantial heterogeneity) and Cochran’s Q test (*p* < 0.10). Prespecified sensitivity analyses evaluated result stability through sequential exclusion of individual studies. Subgroup analyses were stratified by geographical region (eastern, central, western, northeastern China), survey period (before 2015 vs. after 2015), age categories (<26 vs. ≥ 26 years), BMI thresholds (<24 vs. ≥ 24 kg/m^2^), anxiety assessment tools, PCOS diagnostic criteria, study methodological quality (low/moderate/high risk of bias). Meta-regression models evaluated covariates potentially explaining heterogeneity. Robustness of findings was verified through sensitivity analyses using leave-one-out methods. Publication bias was evaluated through visual inspection of funnel plot symmetry and formal testing using Begg’s and Egger’s tests, with *p* < 0.05 indicating potential bias.

## Result

### Study selection and characteristics

**Figure 1:**
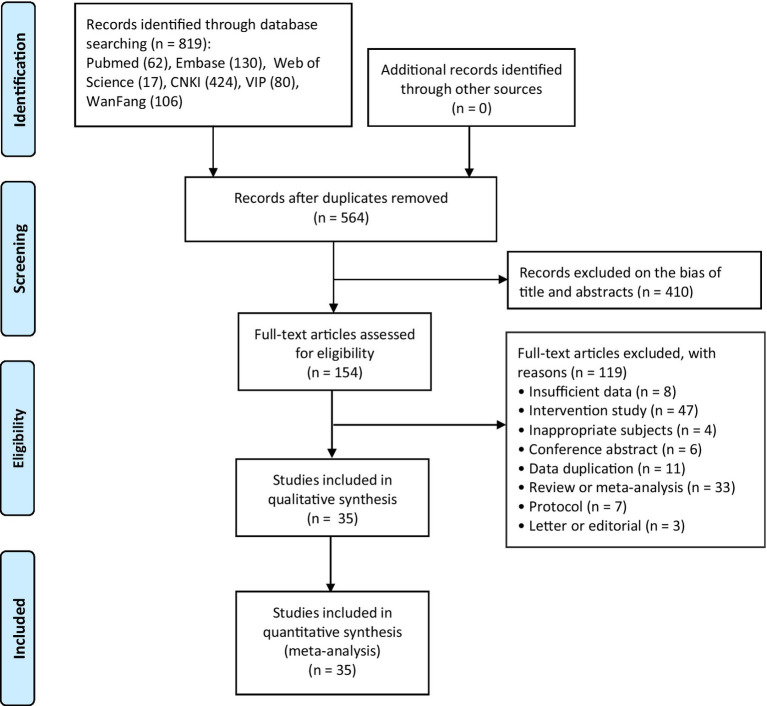
Flow diagram for studies selection.

The systematic search identified 819 records from six databases. After duplicate removal, 564 citations underwent title/abstract screening, with 154 proceeding to full-text review. Thirty-five studies (Yang et al., 2021; Zou, 2014; Zhao, 2020; You, 2022; Ying and Liu, 2023; Yin, 2016; Xue, 2019; Wang et al., 2024; Tan et al., 2020; Tan et al., 2017; Mo et al., 2023; Ma et al., 2013; Feng et al., 2022; Dong et al., 2012; Zhao, 2019; Zhangsun, 2020; Yang, 2020; Wang, 2019; Li, 2011; Li, 2015; Jian, 2020; He, 2021; Hao, 2022; Guo, 2019; Ge, 2008; Deng, 2021; Cheng, 2020; Zhang, 2023; Zhang and Hou, 2019; Zhang and Hou, 2018; Zhang et al., 2010; Yu et al., 2024; Xia et al., 2022; Chen, 2020; Sun and Yi, 2024; *n* = 8,655 participants) met inclusion criteria (Figure 1).

Studies spanned 14 provincial-level regions, categorized by China’s National Bureau of Statistics as: West China (13 studies), East China (12 studies), Central China (5 studies), Northeast China (3 studies). One multicenter study included participants from 29 provinces. Data collection occurred between 2006 and 2024, with publication dates ranging from 2008 to 2024. Mean participant age ranged from 15 to 49 years, BMI ranged 21–27 kg/m^2^. Sample sizes per study varied from 96 to 710 (pooled total: 8655). Anxiety assessment tools contained Self-Rating Anxiety Scale (SAS), followed by Hospital Anxiety and Depression Scale (HADS), Generalized Anxiety Disorder-7 (GAD-7), Beck Anxiety Inventory (BAI), Hamilton Anxiety Scale (HAMA), Symptom Checklist-90 (SCL-90), and State–Trait Anxiety Inventory (STAI). PCOS diagnosis criteria contained Rotterdam criteria (22 studies), People’s Republic of China Health Industry Standards (PRCHIS; 9 studies), and combined criteria (4 studies). Thirty-one studies were published in Chinese, four studies were published in English. Full study characteristics appear in Table 1.

**Table 1:** Characteristic of included studies.

Study (Year)	Publication language	Region	Survey period	Age (years, mean ± sd or range)	BMI (kg/m2, mean ± sd or range)	Anxiety screening tool	PCOS diagnostic criteria	Number of anxiety	Number of PCOS	Prevalence of anxiety in PCOS
Zou, (2014)	Chinese	Sichuan	2013–2014	26.32 ± 4.01	22.29 ± 3.91	SAS	PRCHIS + Rotterdam	42	148	28.38%
Guo, (2019)	Chinese	Shanghai	2016–2018	26.88 ± 4.39	25.24 ± 6.02	SCL90	Rotterdam	30	111	27.03%
Ge (2008)	Chinese	Shandong	2006–2007	27.6 ± 3.5	24.4 ± 4.1	SAS	Rotterdam	49	292	16.78%
Dong et al. (2012)	Chinese	Shanghai	2009–2010	31.79	NR	SAS	Rotterdam	22	128	17.19%
Ma et al. (2013)	Chinese	Shanghai	2009–2010	24.9 ± 5	24.63–25.52	SAS	Rotterdam	15	100	15.00%
Tan et al. (2020)	Chinese	Guangdong	2017–2019	18–45	22.072 ± 4.558	GAD⁃7	Rotterdam	80	262	30.53%
Li (2015)	Chinese	Sichuan	2013–2015	23.75 ± 4.1	21.65 ± 2.89	SAS	PRCHIS + Rotterdam	76	121	62.81%
Wang et al. (2024)	English	Hunan	2022	27.22	22.77	GAD⁃7	Rotterdam	246	477	51.57%
Tan et al. (2017)	English	Sichuan	2013–2014	24.8 ± 3.8	21.4 ± 3.0	STAI	Rotterdam	16	120	13.33%
Yang et al. (2021)	English	Chongqing	2018–2020	28	NR	HADS	Rotterdam	115	433	26.56%
Yin (2016)	Chinese	Tianjin, Heilongjiang	2013–2014	20–40	NR	SAS	Rotterdam	21	115	18.26%
He (2021)	Chinese	Sichuan	2020–2021	21–35	NR	GAD⁃7	PRCHIS + Rotterdam	80	150	53.33%
Zhangsun (2020)	Chinese	Sichuan	2019	24.9 ± 4.0	21.96 ± 3.49	SAS	PRCHIS + Rotterdam	77	214	35.98%
Cheng (2020)	Chinese	Sichuan	2017–2020	25.5 ± 4.7	23.04 ± 4.4	GAD⁃7	PRCHIS + Rotterdam	203	710	28.59%
Hao (2022)	Chinese	Sichuan	2020–2022	20–39	NR	SAS	PRCHIS + Rotterdam	30	138	21.74%
Jian (2020)	Chinese	Sichuan	2018–2019	15–39	21.55 ± 2.65	SAS	PRCHIS	26	126	20.63%
Wang (2019)	Chinese	Shanghai	2018–2019	26.51 ± 5.19	NR	GAD⁃7	Rotterdam	53	117	45.30%
Deng (2021)	Chinese	Chongqing	2018–2020	28	22.79 ± 3.19	HADS	PRCHIS + Rotterdam	115	433	26.56%
Xue (2019)	Chinese	Shanxi	2017–2018	26.35 ± 2.74	25.44 ± 9.2	SAS	PRCHIS	27	130	20.77%
Ying & Liu (2023)	Chinese	Henan	2021–2022	26.75 ± 4.73	27.56 ± 6.87	SAS	Rotterdam	7	138	5.07%
Zhao (2020)	Chinese	Heilongjiang	2018–2019	25.41 ± 5.2	26.6 ± 5.59	SAS	PRCHIS	162	520	31.15%
Li (2011)	Chinese	Guangdong	2009–2011	25.74 ± 4.297	NR	SAS	Rotterdam	64	96	66.67%
Zhao (2019)	Chinese	Shanxi	2017–2018	25.5 ± 4.63	22.82 ± 3.55	SAS	PRCHIS	58	205	28.29%
Feng et al. (2022)	Chinese	Ningxia	2019–2020	25.34 ± 5.37	24.86 ± 4	SAS	Rotterdam	369	448	82.37%
You (2022)	Chinese	Xinjiang	2020–2021	20–40	NR	BAI	Rotterdam	89	200	44.50%
Mo et al. (2023)	Chinese	Beijing	2021–2022	18–45	22.89 ± 4.27	SAS	Rotterdam	43	132	32.58%
Yang (2020)	Chinese	Chongqing	2018–2020	20–49	NR	HADS	Rotterdam	115	433	26.56%
Xia et al. (2022)	Chinese	Shanghai	2019–2022	20–40	NR	SAS	PRCHIS	44	125	35.20%
Zhang & Hou (2018)	Chinese	Heilongjiang	2015–2016	25.02 ± 4.39	21.92 ± 2.02	SAS	Rotterdam	84	222	37.84%
Zhang & Hou (2019)	Chinese	Heilongjiang	2017	25.94 ± 4.60	21.84 ± 1.85	SAS	PRCHIS	99	365	27.12%
Chen (2020)	Chinese	Tianjin	2019–2020	28.40 ± 4.24	NR	HAMA	Rotterdam	54	326	16.56%
Zhang et al (2010)	Chinese	Shanghai	2009–2010	31.79	NR	SAS	Rotterdam	18	128	14.06%
Zhang (2023)	Chinese	Shandong	2021–2022	18–38	NR	SAS	Rotterdam	141	495	28.48%
Yu et al. (2024)	Chinese	Hunan	2024	20–49	NR	GAD-7	Rotterdam	77	210	36.67%
Sun & Yi (2024)	English	29 Provinces	2021	23.55 ± 3.15	NR	HADS	Rotterdam	116	287	40.42%

PCOS, polycystic ovary syndrome; BMI, body mass index; SD, standard difference; NR, not reported; SAS, Self-Rating Anxiety Scale; GAD-7, Generalized Anxiety Disorder-7; STAI, State–Trait Anxiety Inventory; HAMA, Hamilton Anxiety Scale; BAI, Beck Anxiety Inventory; HADS, Hospital Anxiety and Depression Scale; SCL-90, Symptom Checklist-90; PRCHIS, People’s Republic of China Health Industry Standards.

### Meta-analysis

**Figure 2:**
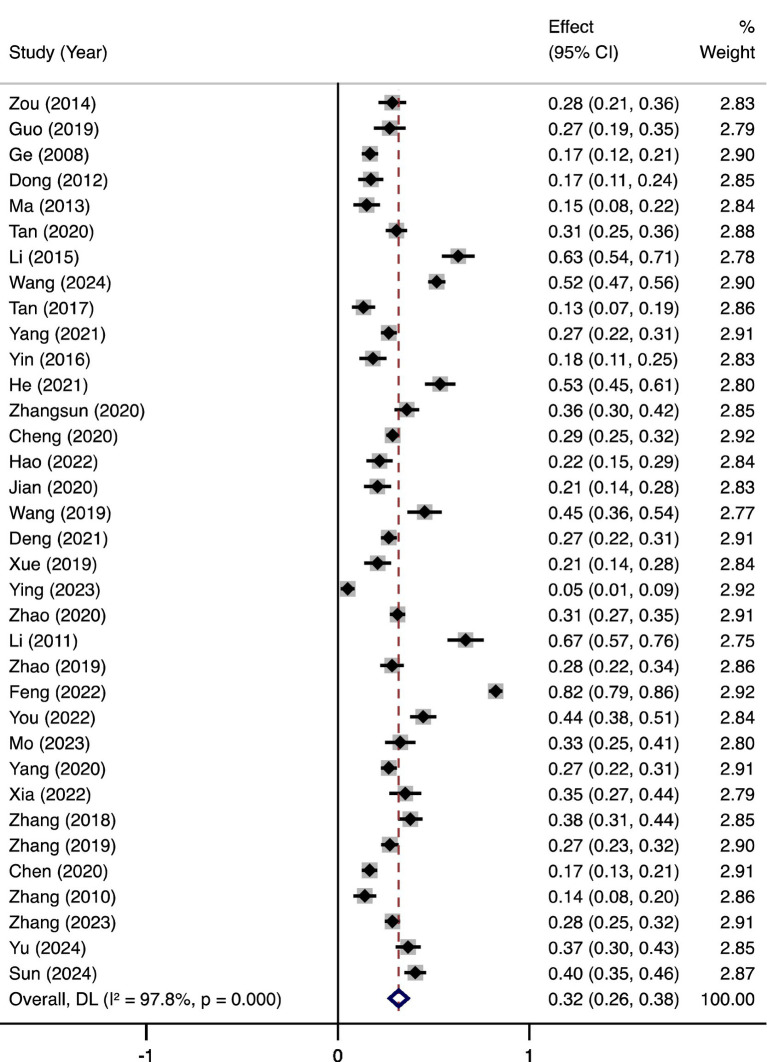
Pooled prevalence of anxiety in women with PCOS.

The pooled prevalence of anxiety among women with polycystic ovary syndrome (PCOS) in mainland China was 32% (95% CI, 26–38%), with substantial heterogeneity (*I*^2^ = 97.8%, *p* < 0.001; Figure 2).

Subgroup analyses were conducted by regions, survey periods, age, BMI, anxiety screening tools, PCOS diagnostic criteria, and study methodological quality.

Anxiety prevalence differed regionally: 36% (95% CI, 24–49%; *I*^2^ = 98.7%) in West China, 28% (95% CI, 22–25%; *I*^2^ = 93.4%) in East China, 28% (95% CI, 9–48%; *I*^2^ = 98.5%) in Central China, and 32% (95% CI, 26–37%; *I*^2^ = 95.4%) in Northeast China (Supplementary Figure 1).

Anxiety prevalence was 28% (95% CI, 17–39%; *I*^2^ = 96%) in studies conducted before 2015, 33% (95% CI, 26–40%; *I*^2^ = 98%) in studies conducted after 2015 (Supplementary Figure 2).

Anxiety prevalence was 39% (95% CI, 26–52%; *I*^2^ = 98.7%) in younger participants (<26 years), 25% (95% CI, 17–32%; *I*^2^ = 96.4%) in those aged ≥26 years (Supplementary Figure 3).

Anxiety prevalence was 32% (95% CI, 26–39%; *I*^2^ = 93.7%) in lower BMI (<24 kg/m^2^) subgroup, 28% (95% CI, 4–52%; *I*^2^ = 99.4%) in higher BMI (≥24 kg/m^2^) subgroup (Supplementary Figure 4).

Anxiety prevalence estimates varied by assessment tool: 31% (95% CI, 21–40%; *I*^2^ = 98.4%) for SAS, 41% (31–50%; *I*^2^ = 94.4%) for GAD-7, and 30% (95% CI, 24–35%; *I*^2^ = 84.5%) for HADS. Only one study used SCL-90, STAI, BAI, and HAMA, respectively, revealing anxiety prevalence of 27, 13, 44, and 17% (Supplementary Figure 5).

Diagnostic criteria also affected estimates, with Rotterdam criteria producing anxiety prevalence of 31% (95% CI, 22–41%; *I*^2^ = 98.5%), PRCHIS 27% (95% CI, 23–31%; *I*^2^ = 63.5%), and combined criteria 36% (95% CI, 28–45%; *I*^2^ = 93.9%; Supplementary Figure 6).

Low-risk studies reported anxiety prevalence of 18% (95% CI, 12–24%; I^2^ = 72.8%), while moderate-risk studies 34% (95% CI, 27–42%; I^2^ = 98.1%), and high-risk study 28% (95% CI, 18–38%; I^2^ = 90.8%; Supplementary Figure 7).

### Sensitivity and meta-regression analysis

Sequential exclusion sensitivity analysis demonstrated no single study exerted disproportionate influence on pooled prevalence estimates (Supplementary Figure 8). Univariate meta-regression evaluating geographic region, survey period, age, BMI, anxiety assessment tools, PCOS diagnostic criteria, and study methodological quality failed to identify significant contributors to heterogeneity (*p* > 0.05 for all covariates; Supplementary Table 2).

### Bias assessment

Funnel plot symmetry, Begg’s regression test (*p* = 0.063) and Egger’s regression test (*p* = 0.86) suggested no potential publication bias (Supplementary Figure 9). Risk-of-bias evaluation classified 4 studies as low risk, 4 studies as high risk, and 27 studies as high risk (Supplementary Table 3).

## Discussion

This systematic review and meta-analysis, encompassing 35 studies and 8,655 participants, is the first to quantify anxiety prevalence among women with PCOS in mainland China. The pooled anxiety prevalence was 32% (95% CI, 26–38%), with substantial heterogeneity (*I*^2^ = 97.8%). Subgroup analyses revealed higher prevalence in younger women, regional disparities (highest in Western China: 36%), and variability by assessment tools (e.g., GAD-7: 41% vs. SAS: 31%). These findings underscore the intersection of biological, psychosocial, and cultural factors driving anxiety in this population.

The observed prevalence aligns with global estimates (37%) reported in recent meta-analyses (Wang et al., 2021), yet contextual differences are critical. In China, anxiety in PCOS may be exacerbated by unique sociocultural stressors, including stigma surrounding infertility, familial pressures for marriage, and body image dissatisfaction amplified by societal beauty standards (Qiu et al., 2020; Xue et al., 2024). Hyperandrogenism and insulin resistance, hallmarks of PCOS, may further dysregulate hypothalamic–pituitary–adrenal axis activity, fostering neuroendocrine pathways linked to anxiety (Sarkisian et al., 2023). Younger women, often navigating fertility expectations and career pressures, face compounded psychosocial burdens. Regional disparities likely reflect socioeconomic inequities, for example, limited mental health resources in West China correlate with higher anxiety rates, consistent with studies linking healthcare access to psychological outcomes (Kroenke et al., 2007).

While the pooled prevalence parallels global figures, methodological and cultural distinctions limit direct comparisons. A meta-analysis by Cooney et al. (2017) reported a 41.9% anxiety prevalence in PCOS populations worldwide but included only one study for Chinese population, Dokras, et al’s meta-analysis reported a 20.4% anxiety prevalence in women with PCOS without Chinese participants (Dokras et al., 2012), highlighting the lack of regional representation leading to differences. Cultural factors also modulate risk, obesity and metabolic dysfunction are primary anxiety drivers in Western cohorts (Almhmoud et al., 2024), whereas in China, the risk factors that cause anxiety among Chinese women are more complex and diverse. Work-home conflict (Liu and Hong, 2023), marriage and fertility pressures (Wang et al., 2022), and body image dissatisfaction (Liu et al., 2022) are important factors leading to anxiety.

The substantial heterogeneity (I^2^ = 97.8%) observed in this meta-analysis is common in large-scale prevalence studies and likely stems from multifaceted methodological and clinical drivers. Firstly, the diversity in PCOS diagnostic criteria plays a pivotal role; the broader Rotterdam criteria, which identify additional phenotypes such as normo-androgenic PCOS, typically yield higher prevalence estimates compared to the more stringent PRCHIS standards tailored to the metabolic profiles of Chinese women (Teede et al., 2023). Secondly, our results indicate that instruments like the GAD-7, characterized by high sensitivity for generalized anxiety in medical settings, identified a higher prevalence (41%) than the SAS (31%; Spitzer et al., 2006). Regional disparities in healthcare infrastructure and mental health literacy likely influenced outcomes. Regional socioeconomic disparities between Western and Eastern China underscore the impact of healthcare access and mental health literacy on the psychological outcomes of this population. West China, with fewer specialized clinics, reported higher prevalence, mirroring studies linking regional deprivation to psychological morbidity. Finally, demographic characteristics, particularly younger age and elevated BMI, serve as consistent moderators, reflecting how body image dissatisfaction and metabolic concerns act as chronic stressors that exacerbate psychological distress in specific PCOS subgroups. Younger women, often untreated for PCOS and facing acute fertility concerns, exhibited elevated anxiety, consistent with findings from Nasiri-Amiri et al. (2023). Conversely, higher BMI groups showed lower anxiety prevalence, possibly due to greater metabolic monitoring and clinical support.

For clinicians, these findings advocate for routine anxiety screening in PCOS care, particularly for younger women and those in underserved regions. Culturally adapted interventions—addressing infertility stigma through community education or integrating mental health services into gynecological care. Researchers should prioritize standardized protocols: harmonizing PCOS diagnostic criteria (e.g., adopting Rotterdam globally) and validating anxiety tools in Chinese populations. Future meta-analyses should incorporate mixed-methods data to explore sociocultural mediators (e.g., familial dynamics, healthcare access) and leverage machine learning to disentangle heterogeneity sources.

This study’s strengths include its focus on an underrepresented population and granular subgroup analyses. By integrating data from Chinese databases (e.g., CNKI, WanFang), it mitigates geographic publication bias prevalent in prior syntheses. Sensitivity analyses confirmed stable pooled prevalence, supporting result robustness. However, significant heterogeneity was observed between the included studies. Despite conducting subgroup analyses in accordance with the predefined protocol, the findings remained unable to fully account for the underlying sources of heterogeneity, suggesting potential unmeasured confounding factors or complex interactions beyond the scope of the current analytical framework. Furthermore, the reliance on various self-report scales rather than structured clinical interviews may introduce misclassification bias, potentially overestimating the prevalence of clinical anxiety. These instruments primarily capture ‘anxiety symptoms’ rather than formal ‘anxiety disorders,’ necessitating a cautious interpretation of the pooled results. Future research should utilize standardized diagnostic gold standards to ensure more precise psychiatric characterization in women with PCOS. Most included studies were cross-sectional designed. Temporal relationships between PCOS and anxiety remain unestablished, longitudinal data are needed to infer causality.

## Conclusion

Nearly one-third of Chinese women with PCOS experience anxiety, a prevalence rate significantly shaped by younger age, residence in Western China, and the use of the GAD-7 assessment tool. These findings underscore the need for culturally-sensitive mental health screening and targeted psychological interventions. Clinicians should prioritize early anxiety detection in younger PCOS patients and those in medically underserved regions to improve overall quality of life.

## Data Availability

The original contributions presented in the study are included in the article/Supplementary material, further inquiries can be directed to the corresponding authors.
