# Correction: Anxiety prevalence among women with polycystic ovary syndrome in mainland China: a systematic review and meta-analysis

**DOI:** 10.3389/fpsyg.2026.1834692

**Published:** 2026-03-27

**Authors:** Shanshan Hong, Zhenzhen Hong, Liying Chen, Meiling Liang, Ming Li, Jiawei Qin

**Affiliations:** 1 Department of Obstetrics and Gynecology, Quan Zhou Women's and Children's Hospital, Quanzhou, China; 2 Department of Endocrinology, Quanzhou First Hospital Affiliated to Fujian Medical University, Quanzhou, China; 3 School of Basic Medical Sciences, Hunan University of Medicine, Huaihua, China; 4 Department of Rehabilitation Medicine, Quanzhou First Hospital Affiliated to Fujian Medical University, Quanzhou, China

**Keywords:** anxiety, China, meta-analysis, polycystic ovary syndrome, prevalence

The order of authors in the author list of the published paper was erroneously given as:

[Zhenzhen Hong, Shanshan Hong, Liying Chen, Meiling Liang, Ming Li and Jiawei Qin].

The correct author list reads:

[Shanshan Hong, Zhenzhen Hong, Liying Chen, Meiling Liang, Ming Li and Jiawei Qin].

The original version of this article has been updated.

