# Supplementary material for: Anxiety prevalence among women with polycystic ovary syndrome in mainland China: a systematic review and meta-analysis
Source: Front Psychol. 2026 Mar 11;17:1767567. doi: 10.3389/fpsyg.2026.1767567 (PMC13013291; doi:10.3389/fpsyg.2026.1767567)
Supplement: Supplementary file 1 [file Data_Sheet_1.docx]

Supplement Table 1. Search strategy.

Supplement Table 2. Univariate meta-regression analysis of potential sources of heterogeneity.

Supplement Table 3. Risk of bias of included studies.

Supplement Figure 1. Subgroup analysis based on different study regions.

Supplement Figure 2. Subgroup analysis based on different survey periods.

Supplement Figure 3. Subgroup analysis based on different participants’ age.

Supplement Figure 4. Subgroup analysis based on different participants’ BMI.

Supplement Figure 5. Subgroup analysis based on different anxiety screening tools.

Supplement Figure 6. Subgroup analysis based on different PCOS diagnostic criteria.

Supplement Figure 7. Subgroup analysis based on different studies’ methodological quality.

Supplement Figure 8. Sensitivity analysis of pooled prevalence of anxiety in women with PCOS.

Supplement Figure 9. Funnel plot for publication bias.

Supplement Table 1. Search strategy.

| PubMed | |
| --- | --- |
| #1 | (anxiety[MeSH Terms]) OR (anxiety disorders[MeSH Terms]) OR (angst [Title/Abstract]) OR (nervousness [Title/Abstract]) OR (hypervigilance [Title/Abstract]) OR (social anxiety[Title/Abstract]) OR (anxiety[Title/Abstract]) OR (anxieties[Title/Abstract]) OR (social anxieties[Title/Abstract]) OR (anxiousness[Title/Abstract]) OR (anxiety disorder[Title/Abstract]) OR (disorder anxiety[Title/Abstract]) OR (neurotic anxiety[Title/Abstract]) OR (anxiety neuroses [Title/Abstract]) |
| #2 | (polycystic ovary syndrome[MeSH Terms]) OR (polycystic ovary syndrome[Title/Abstract]) OR (Ovary Syndrome, Polycystic[Title/Abstract]) OR (Syndrome, Polycystic Ovary[Title/Abstract]) OR (PCOS[Title/Abstract]) OR (Polycystic Ovarian Syndrome[Title/Abstract]) OR (Ovarian Syndrome, Polycystic[Title/Abstract]) OR (Polycystic Ovary Syndrome 1[Title/Abstract]) OR (Sclerocystic Ovarian Degeneration[Title/Abstract]) OR (Ovarian Degeneration, Sclerocystic[Title/Abstract]) OR (Sclerocystic Ovary Syndrome[Title/Abstract]) OR (Stein-Leventhal Syndrome[Title/Abstract]) OR (Stein Leventhal Syndrome[Title/Abstract]) OR (Syndrome, Stein Leventhal[Title/Abstract]) OR (Sclerocystic Ovaries[Title/Abstract]) OR (Ovary, Sclerocystic[Title/Abstract]) OR (Sclerocystic Ovary[Title/Abstract]) |
| #3 | (China[MeSH Terms]) OR (China)) OR (Chinese) |
| #4 | #1 AND #2 AND #3 |
| Embase | |
| #1 | 'anxiety' /exp OR 'anxiety disorder' /exp OR 'anxiety assessment' /exp OR 'anxiety':ab,ti OR 'angst':ab,ti OR 'nervousness':ab,ti OR 'hypervigilance':ab,ti OR 'social anxiety':ab,ti OR 'anxieties':ab,ti OR ' anxiousness':ab,ti OR 'anxiety disorder':ab,ti OR 'anxiety assessment':ab,ti |
| #2 | 'ovary polycystic disease' /exp OR 'polycystic ovary syndrome ':ab,ti OR 'ovary polycystic disease':ab,ti OR 'PCOS':ab,ti OR 'Sclerocystic Ovarian Degeneration':ab,ti OR 'Ovarian Degeneration':ab,ti OR 'Sclerocystic Ovary Syndrome':ab,ti OR 'Stein-Leventhal Syndrome':ab,ti OR 'Stein Leventhal Syndrome':ab,ti OR 'Sclerocystic Ovaries':ab,ti |
| #3 | 'China' /exp OR 'Chinese' /exp OR 'China':ab,ti OR 'Chinese':ab,ti |
| #4 | #1 AND #2 AND #3 |
| Web of Science | |
| #1 | TS=(anxiety) OR TS=(anxiety disorder) OR TS=(angst) OR TS=(nervousness) OR TS=(hypervigilance) OR TS=(social anxiety) OR TS=(anxieties) OR TS=(anxiousness) OR TS=(neurotic anxiety) OR TS=(anxiety neuroses) OR TS=(anxiety assessment) |
| #2 | TS=(polycystic ovary syndrome) OR TS=(PCOS) OR TS=(Polycystic Ovarian Syndrome) OR TS=(Sclerocystic Ovarian Degeneration) OR TS=(Sclerocystic Ovary Syndrome) OR TS=(Stein-Leventhal Syndrome) OR TS=(Stein Leventhal Syndrome) OR TS=(Sclerocystic Ovaries) OR TS=(Sclerocystic Ovary) OR TS=(ovary polycystic disease) |
| #3 | TS=(China) OR TS=(Chinese) |
| #4 | #1 AND #2 AND #3 |
| China National Knowledge Infrastructure | |
| #1 | SU=(焦虑+焦虑症+焦虑症状+焦虑感+焦虑水平+焦虑状态+焦虑障碍) |
| #2 | SU=(多囊+多囊卵巢+多囊卵巢综合症+PCOS) |
| #3 | #1 AND #2 |
| Wanfang database | |
| #1 | 题名或关键词=(“焦虑” OR “焦虑症” OR “焦虑症状” OR “焦虑感” OR “焦虑水平” OR “焦虑状态” OR “焦虑障碍”) |
| #2 | 题名或关键词=(“多囊” OR “多囊卵巢” OR “多囊卵巢综合症” OR “PCOS” ) |
| #3 | #1 AND #2 |
| VIP database | |
| #1 | M=(焦虑 OR 焦虑症 OR 焦虑症状 OR 焦虑感 OR 焦虑水平 OR 焦虑状态 OR 焦虑障碍) |
| #2 | M=(多囊 OR 多囊卵巢 OR 多囊卵巢综合症 OR PCOS ) |
| #3 | #1 AND #2 |

Supplement Table 2. Univariate meta-regression analysis of potential sources of heterogeneity.

| Heterogeneity factors | Coef. | Std. Err. | t | p | 95%CI | Tau2 | I-squared_res | Adj R-squared |
| --- | --- | --- | --- | --- | --- | --- | --- | --- |
| Geographical regions | 0.0273142 | 0.0279443 | 0.98 | 0.336 | -0.0296785, 0.0843069 | 0.02667 | 97.78% | 0.03% |
| Survey periods | 0.0537683 | 0.0638119 | 0.84 | 0.406 | -0.0100896, 0.1835946 | 0.02596 | 97.72% | -0.64% |
| Age | 0.1445868 | 0.0715139 | 2.02 | 0.056 | -0.0037241, 0.2928976 | 0.02972 | 98.09% | 12.16% |
| BMI | -0.0416271 | 0.0846243 | -0.49 | 0.629 | -0.2194161, 0.1361619 | 0.0317 | 98.57% | -4.34% |
| Depression assessment | -0.0021717 | 0.0189138 | -0.11 | 0.909 | -0.0406521, 0.0363087 | 0.0266 | 97.80% | -3.12% |
| PCOS diagnostic criteria | 0.0470464 | 0.0455137 | 1.03 | 0.309 | -0.0455519, 0.1396448 | 0.02578 | 97.82% | 0.06% |
| Methodological quality | 0.0508513 | 0.0581323 | 0.87 | 0.388 | -0.0674197, 0.1691222 | 0.02598 | 97.79% | -0.73% |

Supplement Table 3. Risk of bias of included studies.

| Author (year) | External Validity Items | | | | Internal Validity Items | | | | | | Overall  Score | Overall Risk of Bias |
| --- | --- | --- | --- | --- | --- | --- | --- | --- | --- | --- | --- | --- |
|  | 1 | 2 | 3 | 4 | 5 | 6 | 7 | 8 | 9 | 10 |  |  |
| Zou (2014） | 1 | 1 | 0 | 0 | 1 | 1 | 1 | 1 | 1 | 1 | 8 | Moderate |
| Guo (2019) | 1 | 0 | 0 | 0 | 1 | 0 | 1 | 1 | 1 | 1 | 6 | Moderate |
| Ge (2008) | 1 | 1 | 0 | 0 | 0 | 1 | 1 | 1 | 1 | 1 | 7 | Moderate |
| Dong (2012) | 1 | 1 | 0 | 0 | 1 | 1 | 1 | 1 | 1 | 1 | 8 | Moderate |
| Ma (2013) | 1 | 1 | 0 | 0 | 1 | 1 | 1 | 1 | 1 | 1 | 8 | Moderate |
| Tan (2020) | 1 | 1 | 0 | 0 | 0 | 1 | 1 | 1 | 1 | 1 | 7 | Moderate |
| Li (2015) | 0 | 0 | 0 | 0 | 1 | 1 | 1 | 1 | 1 | 1 | 6 | Moderate |
| Wang (2024) | 1 | 0 | 0 | 0 | 1 | 1 | 1 | 1 | 1 | 1 | 7 | Moderate |
| Tan (2017) | 1 | 1 | 0 | 0 | 1 | 1 | 1 | 0 | 0 | 1 | 6 | Moderate |
| Yang (2021) | 1 | 1 | 1 | 0 | 1 | 1 | 1 | 1 | 0 | 1 | 8 | Moderate |
| Yin (2016) | 1 | 1 | 1 | 0 | 0 | 1 | 1 | 1 | 1 | 1 | 8 | Moderate |
| He (2021) | 1 | 0 | 0 | 0 | 1 | 1 | 1 | 1 | 1 | 1 | 7 | Moderate |
| Zhangsun (2020) | 1 | 0 | 0 | 1 | 1 | 1 | 1 | 1 | 0 | 0 | 6 | Moderate |
| Cheng (2020) | 1 | 0 | 0 | 1 | 1 | 1 | 1 | 1 | 1 | 0 | 7 | Moderate |
| Hao (2022) | 1 | 0 | 0 | 1 | 1 | 1 | 1 | 1 | 1 | 1 | 8 | Moderate |
| Jian (2020) | 1 | 0 | 0 | 0 | 1 | 1 | 1 | 1 | 1 | 1 | 7 | Moderate |
| Wang (2019) | 1 | 1 | 0 | 0 | 1 | 1 | 1 | 1 | 0 | 1 | 7 | Moderate |
| Deng (2021) | 0 | 0 | 0 | 0 | 1 | 1 | 1 | 1 | 1 | 1 | 6 | Moderate |
| Xue (2019) | 0 | 0 | 0 | 0 | 1 | 1 | 0 | 1 | 1 | 1 | 5 | High |
| Ying (2023) | 1 | 0 | 0 | 0 | 1 | 1 | 1 | 1 | 0 | 1 | 6 | Moderate |
| Zhao (2020) | 1 | 1 | 0 | 0 | 1 | 1 | 1 | 1 | 1 | 0 | 7 | Moderate |
| Li (2011) | 1 | 1 | 0 | 0 | 1 | 1 | 1 | 1 | 1 | 1 | 8 | Moderate |
| Zhao (2019) | 1 | 1 | 0 | 0 | 1 | 1 | 1 | 1 | 0 | 1 | 7 | Moderate |
| Feng (2022) | 1 | 0 | 0 | 0 | 1 | 1 | 0 | 1 | 1 | 1 | 6 | Moderate |
| You (2022) | 1 | 0 | 1 | 1 | 1 | 0 | 1 | 1 | 1 | 0 | 7 | Moderate |
| Mo (2023) | 1 | 1 | 0 | 1 | 1 | 1 | 1 | 1 | 1 | 1 | 9 | Low |
| Yang (2020) | 0 | 1 | 0 | 1 | 1 | 1 | 1 | 1 | 1 | 1 | 8 | Moderate |
| Xia (2022) | 0 | 0 | 0 | 1 | 1 | 0 | 1 | 1 | 1 | 1 | 6 | Moderate |
| Zhang (2018) | 1 | 1 | 0 | 1 | 1 | 0 | 1 | 1 | 1 | 1 | 8 | Moderate |
| Zhang (2019) | 1 | 0 | 0 | 1 | 1 | 0 | 1 | 1 | 1 | 1 | 7 | Moderate |
| Chen (2020) | 1 | 1 | 0 | 1 | 1 | 1 | 1 | 1 | 1 | 1 | 9 | Low |
| Zhang (2010) | 1 | 1 | 0 | 0 | 0 | 1 | 1 | 1 | 1 | 1 | 7 | Moderate |
| Zhang (2023) | 1 | 1 | 0 | 0 | 1 | 1 | 1 | 0 | 1 | 1 | 7 | Moderate |
| Yu (2024) | 1 | 1 | 0 | 1 | 1 | 1 | 1 | 1 | 1 | 1 | 9 | Low |
| Sun (2024) | 1 | 1 | 0 | 1 | 0 | 1 | 1 | 1 | 1 | 1 | 8 | Moderate |

Note: 0-No, 1-Yes, item 1 = target population representation risk; item 2 = sample frame representation risk; item 3 = sample random selection risk; item 4 non-response risk; item 5 = data collection risk; item 6 = case definition risk; item 7 = instrument validity and reliability risk ; item 8 = data collection consistency risk; item 9 = prevalence period risk; item 10 = appropriate parameter risk; item 11 = overall risk.

Supplement Figure 1. Subgroup analysis based on different study regions.

Supplement Figure 2. Subgroup analysis based on different survey periods.

Supplement Figure 3. Subgroup analysis based on different participants’ age.

Supplement Figure 4. Subgroup analysis based on different participants’ BMI.

Supplement Figure 5. Subgroup analysis based on different anxiety screening tools.

Supplement Figure 6. Subgroup analysis based on different PCOS diagnostic criteria.

Supplement Figure 7. Subgroup analysis based on different studies’ methodological quality.

Supplement Figure 8. Sensitivity analysis of pooled prevalence of anxiety in women with PCOS.

Supplement Figure 9. Funnel plot for publication bias.
